# Myxolipoma in the tongue - A clinical case report and review of the literature

**DOI:** 10.1186/1758-3284-3-50

**Published:** 2011-12-20

**Authors:** Shigehiro Ono, Majeed Rana, Masaaki Takechi, Ikuko Ogawa, Gaku Okui, Yoshitsugu Mitani, Nils-Claudius Gellrich, Nobuyuki Kamata

**Affiliations:** 1 Department of Oral and Maxillofacial Surgery, Division of Cervico-Gnathostomatology, Graduate School of Biomedical Sciences, Hiroshima University, Japan; 2 Center of Oral Clinical Examination, Hiroshima University Hospital, Kasumi 1-2-3, Minami-ku, Hiroshima, Japan; 3 Department of Cranio-Maxillo-Facial Surgery, Hannover Medical School, Germany

**Keywords:** Myxolipoma, Tongue benign tumor, Lipoma

## Abstract

In this article, we present our experience with a case of myxolipoma of the tongue.

Lipoma is a mesenchymal benign tumor occurring with relatively high frequency. However, myxolipoma, one of the histological variant of lipoma characterized by mature adipose tissue and abundant mucoid substances, in the oral cavity is quite rare.

The patient was a 52-year-old man who noticed a painless mass on the left border of tongue about 2 years ago. The lesion was noted at a complete medical checkup, and the patient was admitted to our institution for detailed examination. The mass was a palpable, soft and elastic nodule, 15 mm in diameter, covered with normal mucosa in the left inferior aspect of the tongue. The border of the tumor was well-defined, and computed tomography (CT) revealed a fat density within the mass. On the basis of these finding, the tumor was clinically diagnosed as lipoma and was excised under general anesthesia. Histopathologically, the tumor was a well-defined lobulated mass surrounded by a thin fibrous capsule within the muscle of the tongue. The tumor was diagnosed as myxolipoma because it was consisted of solid proliferation of mature adipocytes replaced by abundant mucoid substances. The post operative course was uneventful, and there was no evidence of recurrence 4 years after surgery.

## Background

Lipoma is the most common neoplasms arising from fat tissue. They are usually having the character of slow-growing, soft and silent masses. Angiolipoma, spindle cell lipoma, myelolipoma, chondorolipoma and myolipoma are some histologic variants of lipomas. Myxolipoma is a lipoma admixed with abundant mucoid substances and is considered to be a lipoma with a high degree of myxoid change [1,2].

However mixolipoma is infrequently observed as neoplasm in the trunk, it rarely occurs in oral region. Only fourteen cases of involving the oral regions, including tougue, buccal mucosa, and lowerlip have been reported in the English literature [1-4]. The incidence rate in the tongue, the buccal mucosa and the lower lip was almost equal. In this report, the authors present the clinical and histological features of mixolipoma excised from the tongue with review of the literature.

## Case report

A 52-year-old man was referred for a painless mass of the left lateral tongue. He had been aware of the mass for about 2 years previously, but had not sought medical attention. Three months before his first visit, he was found to have this mass at a general checkup. There is nothing special in his previous medical or familial history.

**Figure 1:**
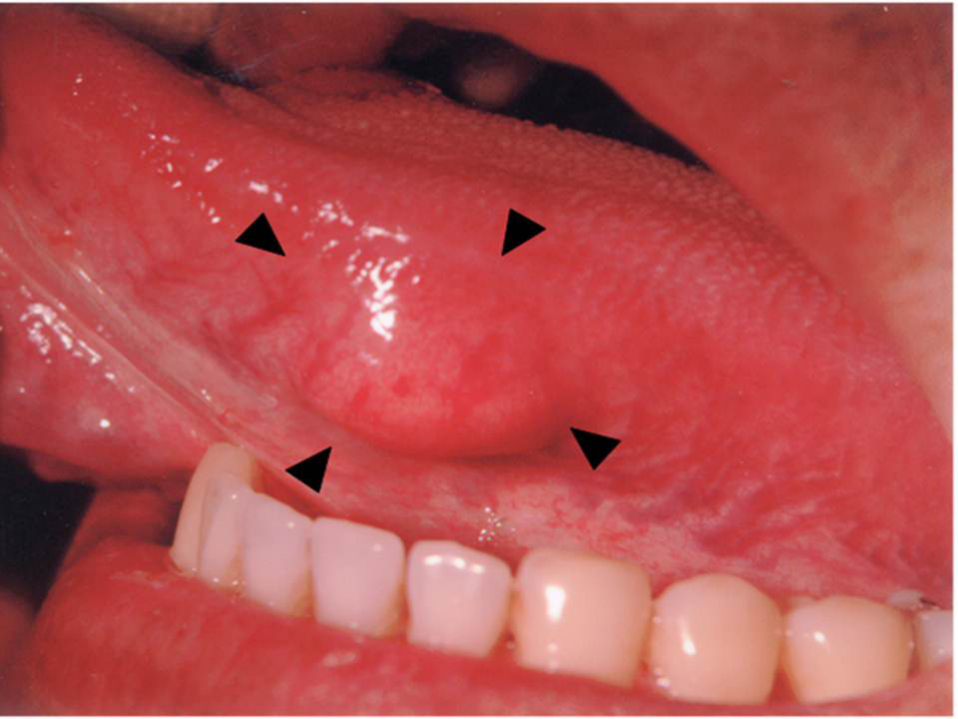
**Clinical photograph of the lesion on the margin of the tongue (anterior surface, 15 × 15 mm)**.

Intraoral examination revealed a solitary, 15 × 15 mm-sized mass on the left border of the tongue (Figure 1). The mass covered with normal mucous membrane was elastic, soft, and movable. Its circumference was not indurated, and the patient felt no tenderness or spontaneous pain. External to the oral cavity, his face was symmetrical, and there were no swollen lymph nodes or tenderness in the neck.

**Figure 2:**
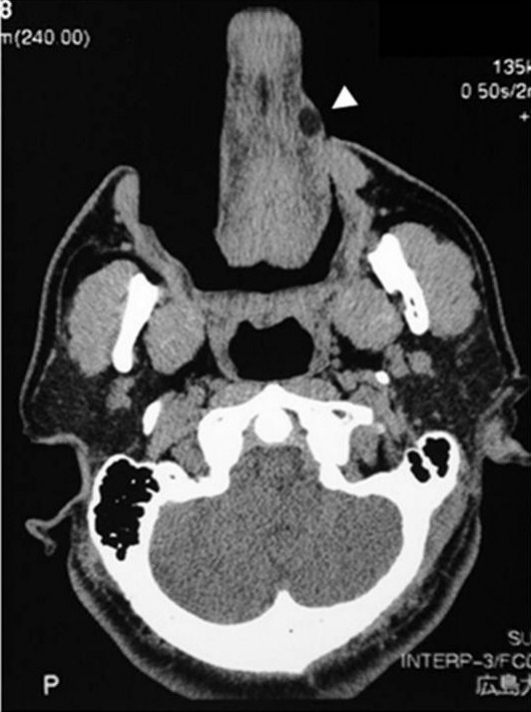
**CT revealed a mass at the left side and the edge of the tongue**.

**Figure 3:**
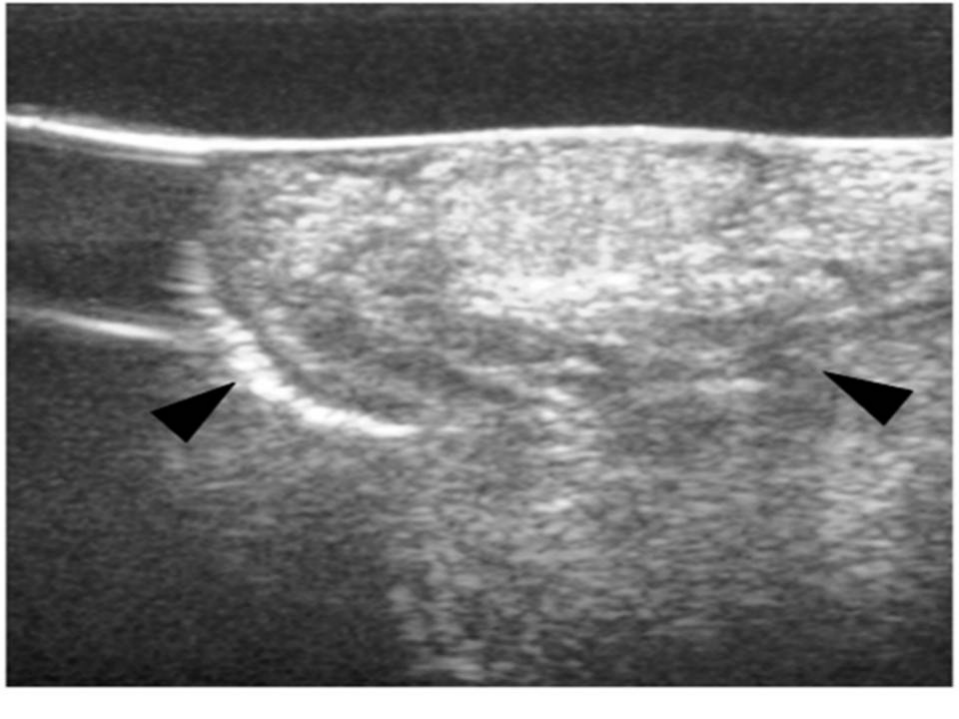
**Echography showed a 15 × 13 × 8 mm dense highly echoic tumor**.

CT showed a 15 mm-sized well-defined lesion with fat concentration inside on the left border of the tongue (Figure 2). Echography showed a 15 × 13 × 8 mm dense highly echoic tumor (Figure 3).

On the basis of these findings, the tumor was clinically diagnosed as a benign lingual tumor (lipoma) and was excised under general anesthesia. The mass was well demarcated and was easily separated from the surrounding tissue.

**Figure 4:**
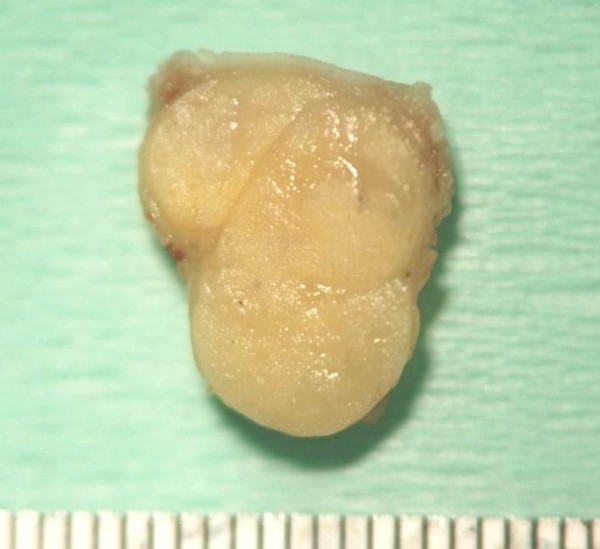
**The surgical specimen measured 1.4 cm in its greatest dimension**.

**Figure 5:**
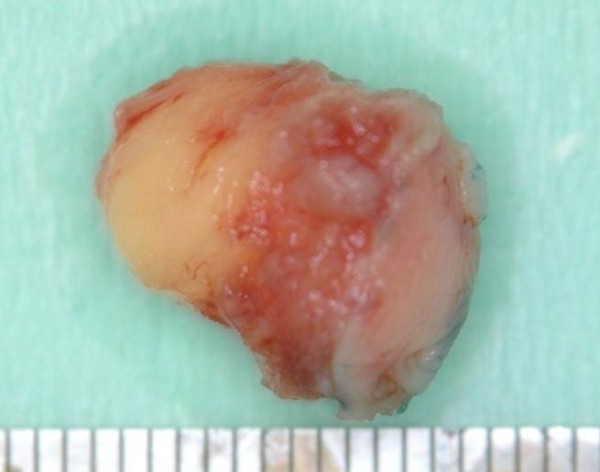
**Cut surface was yellowish-white and lobulated**.

**Figure 6:**
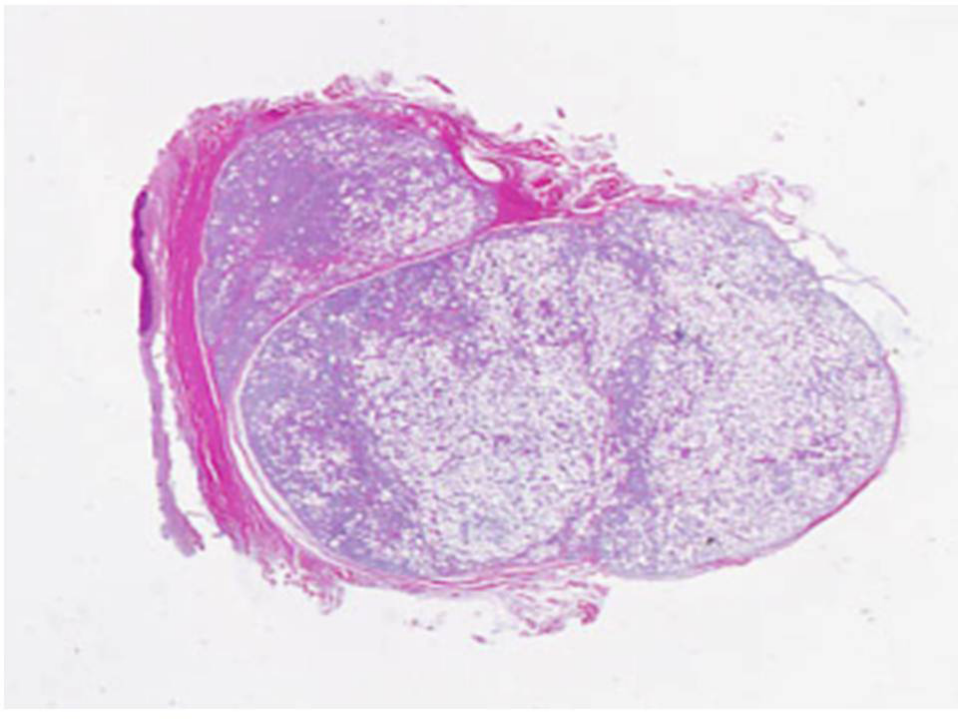
**Photomicrograph of the tumor was encapsulated and lobulated by a thin fibrous tissue (a)**.

**Figure 7:**
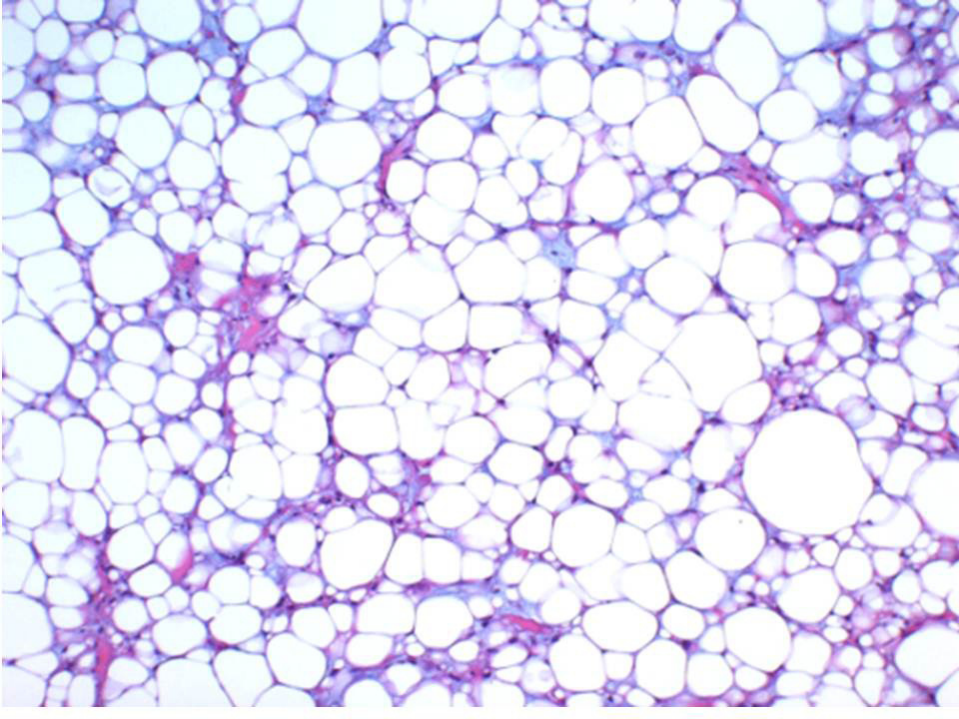
**It was composed of solid proliferation of mature adipocytes**.

**Figure 8:**
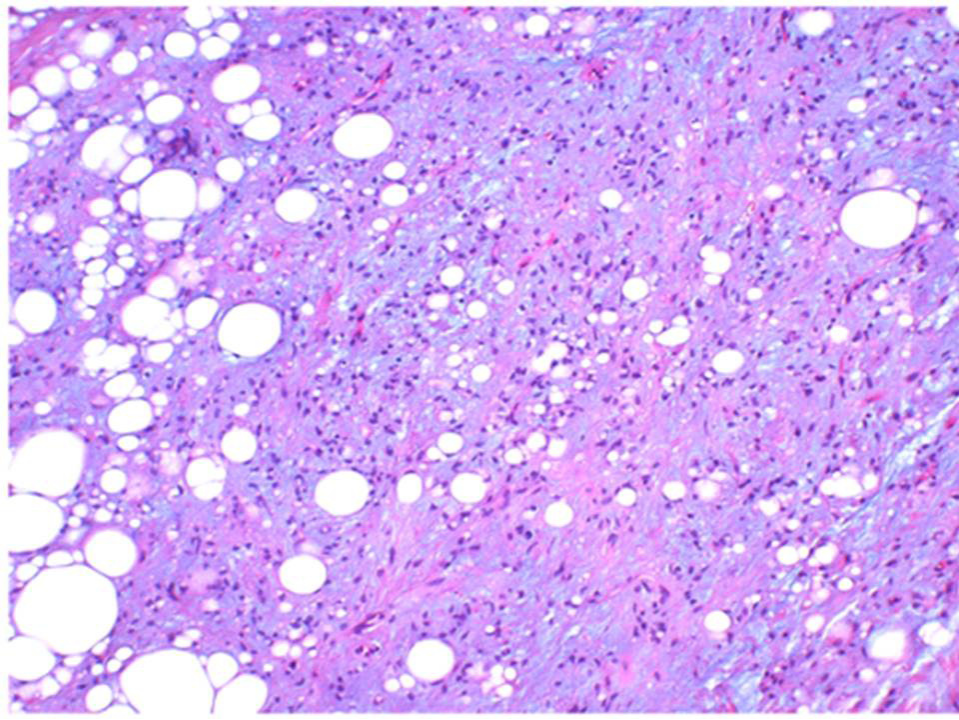
**The solid proliferation replaced by abundant basophilic mucoid substances containing short-spindle smaller cells**.

**Figure 9:**
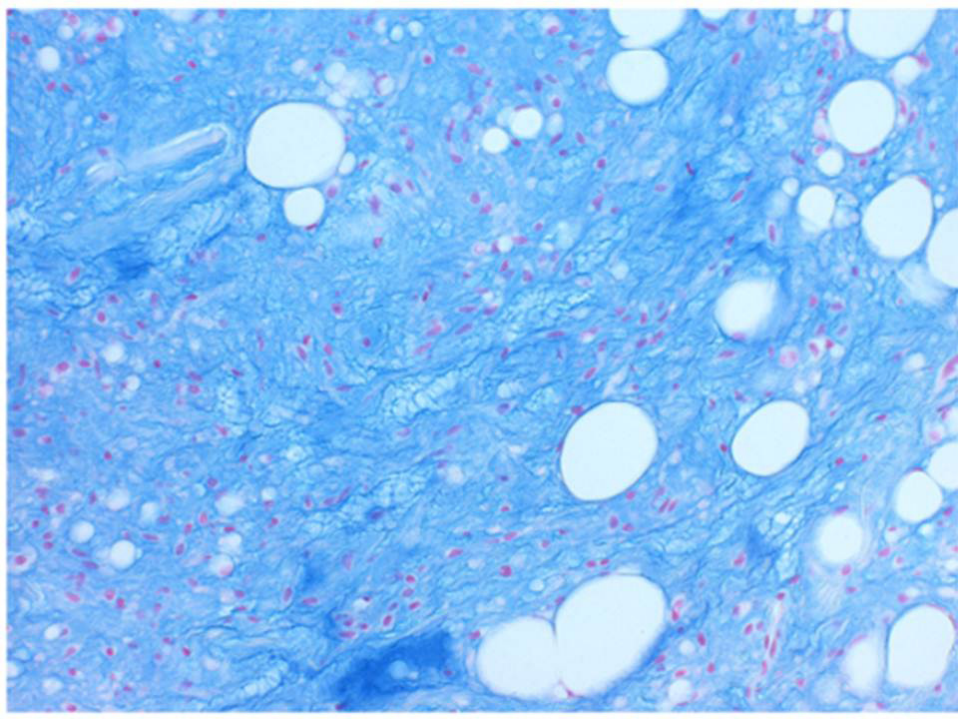
**The matrix showed strong positivity with alcian-blue**.

**Figure 10:**
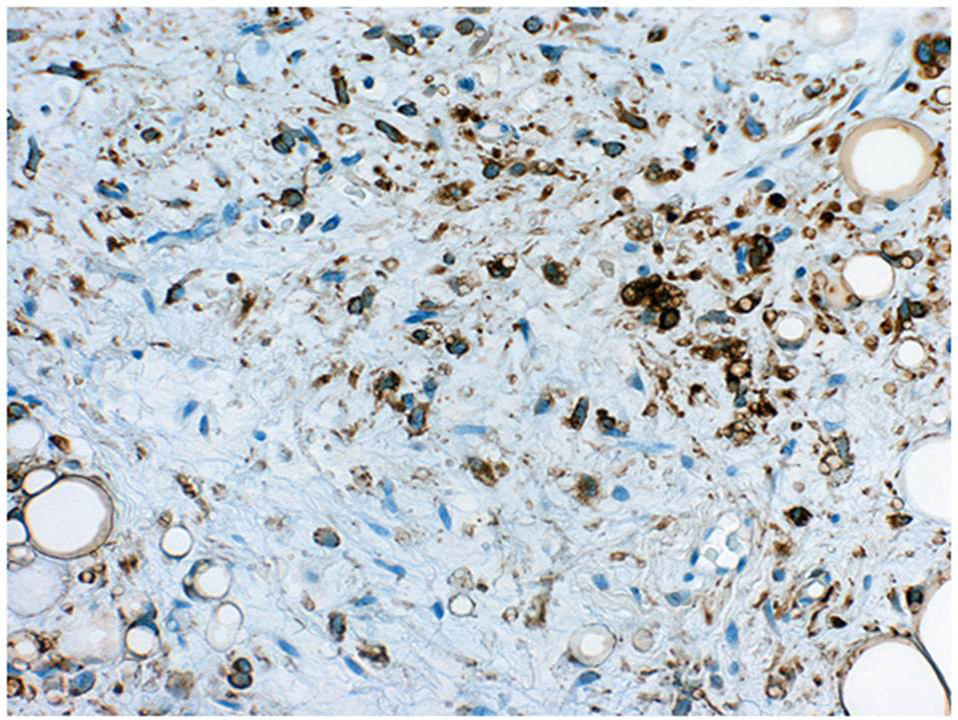
**Immunohistochemical staining patterns**. The mature adipocytes and the shirt-spindle smaller cells were strongly positive for S-100 protein

**Figure 11:**
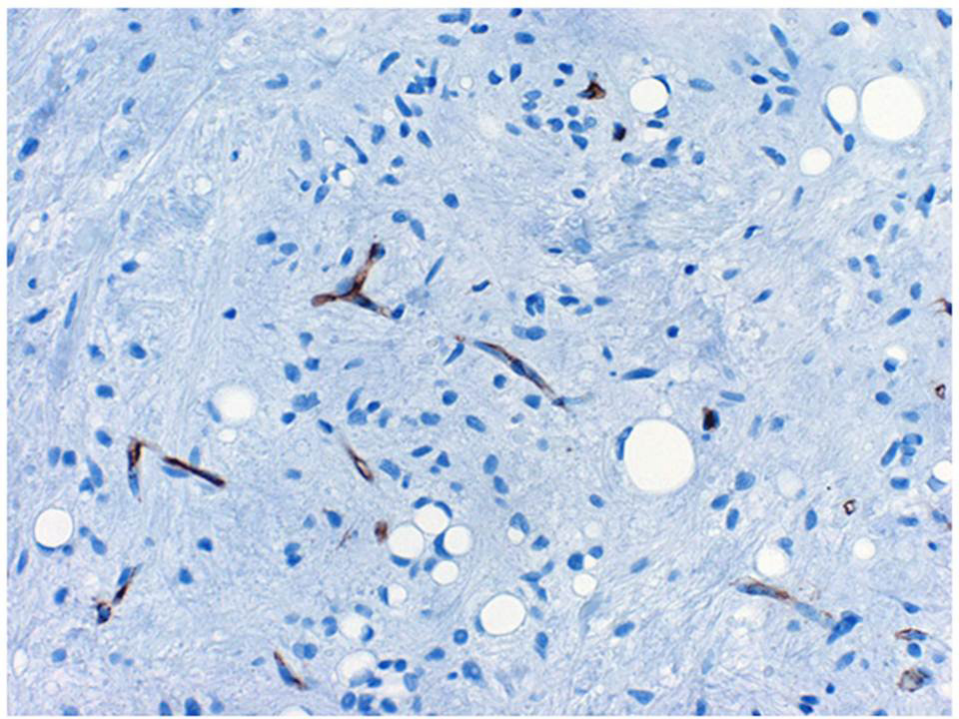
**The mature adipocytes and the shirt-spindle smaller cells were negative for CD34**. And endothelial cells were positive for CD34.

**Figure 12:**
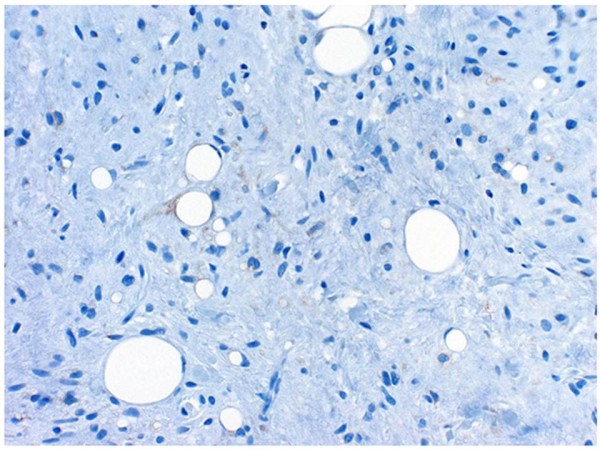
**Immunohistochemical staining patterns showed that the mature adipocytes and the shirt-spindle smaller cells were negative for bcl-2**.

The excised mass was elastic-soft and yellowish-white and showed lobulation on its cut surface (Figure 4, 5). Histopathologically, the tumor was encapsulated and lobulated by a thin fibrous tissue (Figure 6). It was composed of solid proliferation of mature adipocytes admixed with abundant mucoid substances positively stained with alcian blue (Figs. 7, 8, 9). The myxoid areas contained scattered short-spindle smaller cells (Figure 8). Lipoblasts were not present and cellular atypia and mitotic figures were not found. A diffuse plexiform capillary network was not prominent. Immunohistochemically, the tumor cell expressed S-100 protein and were negative for CD34 and bcl-2 (Figure 10, 11, 12). Final diagnosis of myxolipoma was made.

Postoperatively, there has been no lingual dysfunction or any particular problems. The post operative course was uneventful, and there was no evidence of recurrence 4 years after surgery.

## Discussion

Although lipoma represents the most common mesenchymal tumor, its occurrence in the oral and maxillofacial regions is not frequent. As lipomas are occasionally altered by an admixture of other mesenchymal elements, the microscopic variants have been described, including fibrolipoma, sclerotic lipoma, chondrolipoma, osteolipoma, myolipoma and angiomyxolipoma. Myxolipoma is a lipoma admixed with abundant mucoid substances and is considered to be a lipoma with a high degree of myxoid change[1,2]. The mucoid substances are positively stained with alcian blue and are digested by hyaluronidase. Only 14 cases of this variant involving the oral regions have been reported in the English literature (Table 1). Ages ranged from 30 to 70 years The incidence rate in the tongue, the buccal mucosa and the lower lip was almost equal.

**Table 1:** Clinical feature of reported patients with Myxolipoma in oral cavity

Patient's ID	Clinical feature				
	
	***age/sex***	***site***	***treatment***	***progress***	***author***
1	52 male	tongue	resection	unknown	Auband JR[1]
2	70 female	tongue	resection	no relapse	Chen[2]
3	42 male	tongue	resection	no relapse	
4	30 male	buccal mucosa	resection	no relapse	
5	57 male	buccal mucosa	resection	no relapse	
6	unknown	buccal mucosa	resection	no relapse	Said-Al-Naief N[3]
7	unknown	tongue	resection	no relapse	
8	unknown	tongue	resection	no relapse	
9	unknown	lower lip	resection	no relapse	
10	unknown	lower lip	resection	no relapse	
11	unknown	lower lip	resection	no relapse	
12	unknown	lower lip	resection	no relapse	
13	unknown	gingiva	resection	no relapse	
14	55 male	buccal mucosa	resection	no relapse	Studart-Soares EC[4]

Myxolipoma has to be distinguished from benign and malingnant lipomatous tumors with abundant mucoid substances, such as chondroidlipoma, spindle cell lipoma with myxoid changes and myxoid liposarcoma. Chondroidlipoma shows nest-like or cord-like growth of lipoblasts, and its mucoid matrix is resistant to hyaluronidase[5]. Spindle cell lipoma with myxoid change includes CD34-positive and bcl-2-positive spindle cells, which develop on endocapillary cells[6]. Myxoid liposarcoma is a malignant tumor that includes atypical lipoblasts and is characterized by a rich capillary network [7]. Clinical and histopathological findings of the present case are summarized as follows. The tumor was encapsulated and lobulated by a thin fibrous tissue and was composed of a mixture of a solid growth of mature adipocytes and areas rich in mucoid substances posotively stained with alcian-blue and digestable by hyaluronidase. Immunohistochemical analysis showed that short spindle cells in mucoid substances in addition to mature adipocytes are positive for S-100 and negative for CD34 and bcl-2 [6]. There were no malignant features including lipoblast and an abundant capillary network [7]. Therefore, we finally diagnosed the present tumor as myxolipoma.

As for treatment, surgical excision becomes first choice [1-4]. There have been no reports of relapse in any of the reports of myxolipma occurring in the entire body, whether within the oral cavity, on the larynx [8], on the thigh [9], on the skin [10], or in the cervicomediastinum [11]; thus, we consider myxolipoma to carry a relatively benign prognosis. In the present case, for the 4 postoperative years until the present, the patient has suffered no relapse, and he is making satisfactory progress.

## Competing interests

The authors declare that they have no competing interests.

## Authors' contributions

SO, MR, MT, IO, GO, YM, NCG and NK conceived of the study and participated in its design and coordination. SO and MR drafted the manuscript and contributed equally to this work. MR, NCG and NK were involved in revising the manuscript. All authors read and approved the final manuscript.

## Consent statement

Written informed consent was obtained from the patient for publication of this case report and accompanying images. A copy of the written consent is available for review by the Editor-in-Chief of this journal.

## Funding

The article processing charges are funded by the Deutsche Forschungsgemeinschaft (DFG), "Open Access Publizieren".
